# Estimated glucose disposal rate predicts frailty through diabetes: Evidence from machine learning and mediation models in NHANES

**DOI:** 10.1371/journal.pone.0333388

**Published:** 2025-10-07

**Authors:** Wentao Yang, Qian Cheng, Guoxin Huang, Hongming Lin

**Affiliations:** 1 Department of Plastic Surgery, Xiangyang No.1 People’s Hospital, Hubei University of Medicine, Xiangyang, Hubei, China; 2 Health Management Center, Affiliated Hospital of Hubei University of Arts and Science Xiangyang Central Hospital, Xiangyang, Hubei, China; 3 Department of Evidence-Based Medicine Center, Xiangyang No.1 People’s Hospital, Hubei University of Medicine, Xiangyang, Hubei, China; National Healthcare Group, SINGAPORE

## Abstract

**Objective:**

As an emerging insulin resistance marker, the relationship between estimated glucose disposal rate (eGDR) and frailty needs further exploration. This study examines the eGDR-frailty link, develops a machine learning predictive model to address this gap, and explores diabetes mellitus (DM) as a mediator, providing new insights for clinical intervention.

**Methods:**

Using National Health and Nutrition Examination Survey (NHANES) 2005–2010 data, we analyzed glucose disposal and frailty associations. Feature selection used LASSO, and class imbalance was handled by SMOTEN. The resampled data were split 7:3 into a training set (n = 29,309) and a test set (n = 12,561).Ten machine learning models were built, with discrimination, calibration, and clinical utility evaluated to identify the optimal model. Confusion matrices visualized performance. Mediation analysis assessed DM’s role in the eGDR-frailty relationship.

**Results:**

Among 26,282 participants, eGDR negatively correlated with frailty. Higher eGDR significantly reduced frailty risk in subgroups: women, age ≤ 60, normal/high BMI, never/current smokers, and alcohol users. LASSO selected 12 predictors. Across 10 models, CatBoost performed best on the test set (AUC = 0.970, accuracy = 0.920, F1 = 0.918), with robust calibration and decision-curve net benefit. SHAP interpretation ranked eGDR among the most influential predictors: SHAP summary and dependence plots indicated that higher eGDR decreased the model’s predicted probability of frailty. Confusion matrices validated classification accuracy. Mediation analysis showed DM partially mediated the eGDR-frailty relationship: indirect effect β=−0.003 (95% CI −0.003 to −0.002; P < 0.001), mediation proportion = 8.71%.

**Conclusion:**

This first NHANES-based study demonstrates a significant negative correlation between eGDR and frailty, confirming DM’s partial mediating role. The developed machine learning models effectively support early frailty risk assessment and intervention.

## Introduction

Frailty is a complex biological syndrome primarily triggered by the gradual decline of multiple physiological systems. Its typical manifestations include a significant decrease in physiological reserve and stress resistance, significantly increasing the health risks for patients [1]. Community-based studies suggest that the prevalence of frailty among the general population is around 13%, with this condition being more prevalent among female residents, and morbidity rate prevalence rising with age [2]. Notably, frailty is not exclusive to the elderly; the young and middle-aged individuals may also be affected [3,4]. The emergence of symptoms is closely associated with various factors, including lifestyle, socioeconomic status, and health behavior and so on [5]. Given the profound impact of frailty on individual functional decline and quality of life, early identification and effective intervention measures are particularly crucial [6]. Currently, many types of assessment tools are utilized for frailty evaluation, among which the Frailty Index (FI) is widely employed for comprehensive assessment of an individual’s frailty level due to its comprehensiveness and accuracy [7,8].

The glucose disposal rate (eGDR) is a critical indicator for assessing the body’s capacity to metabolize and utilize glucose, serving as an effective alternative tool for measuring the degree of insulin resistance (IR). Insulin resistance is characterized by a decreased responsiveness of body cells to insulin, which leads to diminished efficacy in promoting glucose utilization. This pathological state is closely associated with various chronic diseases, including frailty, chronic kidney disease, hepatic steatosis, atherosclerosis, hypertension, and cardiovascular diseases etc [9–12]. Conventional approaches to assessing insulin resistance, like the hyperinsulinemic-euglycemic clamp, are highly accurate but are restricted in clinical settings and large-scale studies because of their complexity, high costs, and invasiveness [13]. In contrast, eGDR has gained widespread use in medical research and clinical practice owing to its simplicity of operation, cost-effectiveness, and non-invasive characteristics [14]. Despite significant advancements in the study of frailty and glucose disposal rate (eGDR) within their respective fields, there remains insufficient exploration regarding the relationship between these two factors.

Previous research results has indicated that there was a certain association between IR and frailty, suggesting that eGDR may also have a potential connection with frailty [15,16]. Recent studies based on the China Health and Retirement Longitudinal Study (CHARLS) and the Health and Retirement Study (HRS) have found that eGDR is negatively correlated with frailty progression, independent of whether individuals have DM [17,18]. Although the aforementioned studies have progressively unveiled the strong link between low eGDR levels and the onset and progression of frailty, there remains a lack of predictive mechanisms for frailty based on eGDR. To address this gap, this study, for the first time, utilizes NHANES data to thoroughly investigate the relationship between eGDR and frailty and constructs machine learning – based predictive models, providing robust tools for early risk assessment and timely intervention of frailty. Additionally, to further explore the role of DM in this process, we employ mediation analysis using the Bootstrap method to examine the mediating effect of DM between eGDR and frailty. We hope that these findings will offer new perspectives for the prevention and treatment of frailty and lay the foundation for future frailty intervention measures, thereby alleviating the global burden of frailty.

## Materials and methods

### Data source

Data utilized in this study were obtained from the National Health and Nutrition Examination Survey (NHANES), which was administered by the National Center for Health Statistics (NCHS) from 2005 to 2020. NHANES is a nationally representative assessment that examines the health and nutritional status of the U.S. population via an extensive array of physical examinations, laboratory analyses, and interviews [19]. Ethical approval for the collection of all data was obtained from the NCHS Institutional Review Board, and Written consent was obtained from all participants. This study utilizes de-identified NHANES data, which are publicly available and permit researchers to conduct secondary analyses without the need for additional ethical approval. The original dataset and the code are shared on the Figshare website.(dataset:10.6084/m9.figshare.29987881;code:10.6084/m9.figshare.29987935).

### Frailty assessment

The FI in this study was constructed following the standard deficit-accumulation approach proposed by Searle et al., in which eligible health deficits are recoded on a 0–1 scale and aggregated as the proportion of deficits present [20]. The FI encompasses 49 specific indicators, including cognitive function, self-care ability in daily life, depression, chronic diseases, medical utilization, overall health status, physical function, anthropometric measurements (such as BMI), and laboratory test results. The calculation method of the FI involves accumulating the number of deficits an individual has and apportioning this aggregate by the overall count of potential deficits, resulting in a continuous score. These data were obtained from the NHANES database. Each deficit is quantified within a score range of 0–1, based on its impact on individual function. To more precisely evaluate the frailty status of participants, this study adopted a 0.1-point gradient for grading the FI, thereby categorizing participants into the non-frailty group (FI < 0.2) and the frailty group (FI ≥ 0.2) [21,22].

### eGDR assessment

eGDR is a novel indicator used to assess IR. We utilized readily available clinical parameters from the NHANES database to evaluate eGDR, such as glycated hemoglobin (HbA1c), blood pressure status (hypertension or normal blood pressure), and waist circumference (WC). The specific calculation formula is: eGDR = 21.158 − (0.09 × WC) − (3.407 × HT) − (0.551 × HbA1c), where WC represents waist circumference (in centimeters), HT indicates hypertension status (yes = 1, no = 0), and HbA1c denotes the percentage of glycated hemoglobin [23]. For more detailed methods, please refer to (S1 Table).

### Covariate

Covariates considered in this study included age, gender (male, female), race (white, Mexican American, black, and other), education level (under high school, high school or equivalent, high school or above), poverty status, marital status (married, living with partner, separated, divorced, widowed, never married), body mass index (BMI), and health status (including DM, hypertension, cancer, drinking habits, and smoking status). Physical activity levels were measured by total metabolic equivalents. Depression score (assessed via PHQ-9) and systemic immune-inflammation index (SII) were additionally included.

### Statistical analysis

Cross-sectional analysis was conducted using NHANES data from 2005 to 2020, focusing on the association between eGDR and frailty. The study results were weighted to ensure they represent characteristics of the entire U.S. population. In statistical analysis, continuous variables following a normal distribution were presented as mean ± standard deviation (SD), whereas those with a non-normal distribution were described using median (interquartile range, IQR). Categorical variables were expressed as proportions. Categorical variables are reported using percentages. For normally distributed data, group comparisons were conducted via t-tests, whereas non-parametric tests were applied to data that did not follow a normal distribution. Categorical variables were expressed as percentages and analyzed using Chi-square tests. Given the sample size (n = 26,282; frailty prevalence approximately 20.3%), the number of events per predictor variable exceeded the recommended minimum of 10, ensuring adequate power for model development. The logistic regression analysis incorporated NHANES sample weights and accounted for potential confounders associated with frailty, thereby improving the validity of the study findings. Tertile analysis was conducted to further understand the distribution of eGDR. To minimize multicollinearity and improve model stability, variance inflation factor (VIF) analysis was first conducted to identify and exclude highly collinear variables(S2 Table). Three models were built to analyze the association between eGDR and frailty step by step: a basic unadjusted weighted logistic regression model (Model 1), an adjusted model incorporating age, gender, and race/ethnicity factors (Model 2), and a fully adjusted model with all covariates included (Model 3). Trend tests were also conducted to clarify whether a linear relationship exists between eGDR and frailty. To explore in more detail the association between eGDR and frailty in different subgroups, subgroups were analyzed for gender (male/female), age (≤60 years, > 60 years), BMI (normal, low, high), drinking habits (never, previous, heavy, light, moderate), and smoking status (never, previous, current). All statistical analyses were conducted using R Studio (version 4.2.1), employing two-sided tests with a significance threshold set at p < 0.05.

The remaining variables were standardized and entered into a five-fold cross-validated least absolute shrinkage and selection operator (LASSO) logistic regression to obtain a parsimonious set of predictors. Class imbalance was then addressed using SMOTEN to create a balanced dataset. The balanced data were split 7:3 into a training set and a test set using a fixed random seed. The models were constructed using automated hyperparameter optimization methods to identify the optimal parameter settings. Ten machine-learning algorithms were trained on the selected features: Logistic Regression (LR), Support Vector Machine (SVM), Random Forest (RF), Gradient Boosting Decision Tree (GBM), K-Nearest Neighbors (KNN), Extreme Gradient Boosting (XGBoost), LightGBM, CatBoost, AdaBoost, and Neural Network (NN). During the model development, 5 iterations of 10-fold cross-validation and grid search parameter optimization were conducted to enhance model stability. This repeated cross-validation procedure, combined with hyperparameter tuning, minimized overfitting and enhanced model generalizability. The performance of each model was evaluated on both the training and test sets. Model discrimination was assessed using receiver operating characteristic (ROC) curves and area under the curve (AUC) values, along with calculation of optimal threshold, accuracy, sensitivity, specificity, precision, and F1-score. To evaluate probability calibration, calibration curves were plotted and Brier scores were computed to examine the agreement between predicted probabilities and observed probabilities. Decision curve analysis (DCA) was conducted to quantify the net benefit across clinically relevant threshold ranges, with comparison to baseline strategies for assessing clinical utility. Classification performance was visualized through confusion matrices to demonstrate concordance between predicted and actual classifications. For the optimal performing model, permutation feature importance was calculated to identify key predictive variables for frailty, providing preliminary insights to guide clinical research directions. For the best-performing model on the test set (CatBoost), we conducted SHAP analyses (summary, bar, waterfall, force, and decision plots) to interpret feature.

To investigate whether DM mellitus mediates the relationship between estimated glucose disposal rate (eGDR) and frailty, this study conducted mediation analysis in the full sample. Using DM status as the mediator variable, eGDR as the independent variable, and frailty status as the dependent variable, we employed a bias-corrected nonparametric bootstrap method with 5,000 resamples to estimate the total effect, direct effect, and indirect effect. After adjusting for all covariates, the effects of each pathway were evaluated through standardized β coefficients and 95% confidence intervals (CIs). The significance of the indirect effect was determined by whether the 95% CI excluded zero.

This study was conducted using data from the National Health and Nutrition Examination Survey (NHANES), a publicly accessible database provided by the U.S. Centers for Disease Control and Prevention (https://www.cdc.gov/nchs/nhanes/). The raw dataset (https://doi.org/10.6084/m9.figshare.29987881) and the analysis code (https://doi.org/10.6084/m9.figshare.29987935) supporting the findings of this study are openly available in Figshare, providing full transparency and facilitating reproducibility.As this is a secondary analysis of de-identified data, patients and the public were not involved in the study design, conduct, or reporting.

## Results

### Baseline demographic and clinical characteristics

**Fig 1:**
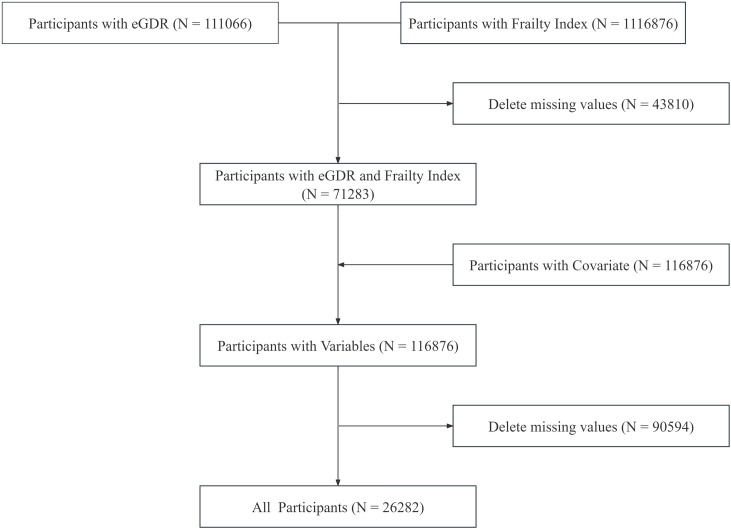
Screening process diagram.

The selection procedure is depicted in Fig 1. A total of 111,066 participants with available eGDR data and 1,116,876 participants with frailty index information were identified from 1990 to 2023. After merging the two datasets and excluding 43,810 participants with missing values, 71,283 participants remained. Subsequently, 116,876 participants with covariate information were included, and 90,594 participants with missing values were excluded, leaving 26,282 participants for the final analysis.

Table 1 shows that after adjusting for all covariates and excluding participants with missing values, the study cohort comprised 26,282participants, featuring a mean age of 45.94 ± 0.25 years, with 52.02% identified as male and the remaining 47.98% as female. Among the non-frail group, the mean age was 44.40 ± 0.25 years, with 54.05% males and 45.95% females; among the frail group, the mean age was 54.37 ± 0.36 years, with 40.93% males and 59.07% females. The two groups exhibited disparities in gender, age, BMI, race, marital status, education level, drinking habits, smoking status, poverty status, physical activity level, hypertension, diabetes, eGDR, cancer, depression score, and SII.

**Table 1:** Weighted selected characteristics of study population in female and male, NHANES (Weighted N = 26282).

Variable	Total	Frality	No-Frailty	Pvalue
Age,year (SD)	45.94(0.25)	44.40(0.25)	54.37(0.36)	< 0.0001
BMI (SD)	28.63(0.08)	28.17(0.09)	31.19(0.16)	< 0.0001
Poverty (SD)	3.30(1.67,5.00)	3.48(1.81,5.00)	2.25(1.16,4.24)	< 0.0001
Physical activity (SD)	1920.00 (720.00,5120.00)	1920.00 (720.00,5280.00)	1440.00 (480.00,4560.00)	< 0.0001
Depression score (SD)	1.00(0.00,4.00)	1.00(0.00, 3.00)	6.00(2.00,11.00)	< 0.0001
SII (SD)	469.23 (342.48,651.26)	462.78 (342.00,633.86)	510.40 (346.65,756.00)	< 0.0001
Sex, %				< 0.0001
Male	52.02	54.05	40.93	
Female	47.98	45.95	59.07	
Race/Ethnicity, %				< 0.0001
White	71.18	71.81	67.74	
Mexican	7.42	7.65	6.2	
Black	9.61	8.7	14.59	
Other	11.78	11.84	11.47	
Marital status, %				< 0.0001
Married	57.73	58.83	51.7	
Living with partner	7.03	7.23	5.94	
Separated	1.86	1.56	3.48	
Divorced	8.69	8.11	11.91	
Widowed	6.07	4.78	13.12	
Never married	18.62	19.49	13.84	
Education, %				< 0.0001
Under high school	12.43	11.27	18.78	
High school or equivalent	22.32	21.16	28.68	
Above high school	65.25	67.57	52.54	
Alcohol intake, %				< 0.0001
Never	8.92	8.92	8.92	
Former	13.24	11.28	24	
Mild	37.16	37.95	32.82	
Moderate	18.21	18.58	16.19	
Heavy	22.46	23.26	18.08	
Smoking, %				< 0.0001
Never	55.01	57.43	41.74	
Former	25.03	23.97	30.8	
Now	19.97	18.6	27.46	
Cancer, %				< 0.0001
No	90.62	92.58	79.91	
Yes	9.38	7.42	20.09	
Diabetes, %				< 0.0001
No	91.6	95.25	71.59	
Yes	8.4	4.75	28.41	
Hypertension, %				< 0.0001
No	65.52	71.27	34.04	
Yes	34.48	28.73	65.96	
eGDR	8.08(0.04)	8.44(0.04)	6.11(0.06)	< 0.0001
eGDRQ				
T1	28.45	23.14	57.57	< 0.0001
T2	34.09	35	29.09	
T3	37.46	41.86	13.34	

### The association between eGDR and frailty

When eGDR becomes a continuous variable, the model 1 results show that eGDR is a protective factor for frailty, with an OR of 0.72 (0.71, 0.73), P-value < 0.0001; the results of model 2 present an OR of 0.74 (0.72, 0.75), P-value < 0.0001; the results of the model 3 results indicate that eGDR is still a protective factor against frailty, with an OR of 0.82 (0.75, 0.89), P-value < 0.0001. When eGDR is the categorical variable, the results of model 1 show that the OR compared to T1, T2 is 0.33 (0.30, 0.37), P-value < 0.0001; for T3, the OR is 0.13 (0.11, 0.15), P-value < 0.0001. The results of model 3 show that the OR compared to T1, T2 is 0.85 (0.68, 1.06), P = 0.15, and for T3, the OR is 0.67 (0.46, 0.96), P = 0.03. Furthermore, there is a linear relationship among the quartiles (P for trend < 0.001). Gernerally, a substantial inverse relationship exists between eGDR and frailty, suggesting that elevated eGDR levels are associated with a reduced probability of frailty. This association was validated in three different models and remained significant in the fully adjusted model for the continuous variable and the highest tertile (T3) (Table 2).

**Table 2:** Weighted ORs (95% CIs) for the associations between betweenc across three models.

Variable	Frailty Index					
	Model 1		Model 2		Model 3	
	OR(95%CI)	P value	OR(95%CI)	P value	OR(95%CI)	P value
eGDR	0.72(0.71,0.73)	<0.0001	0.74(0.72,0.75)	<0.0001	0.82(0.75,0.89)	<0.0001
T1	Ref		Ref		Ref	
T2	0.33(0.30,0.37)	<0.0001	0.37(0.33,0.42)	<0.0001	0.85(0.68,1.06)	0.15
T3	0.13(0.11,0.15)	<0.0001	0.16(0.14,0.19)	<0.0001	0.67(0.46,0.96)	0.03
P for trend		<0.0001		<0.0001		0.03

Model 1: eGDR/eGDRT; Model 2: eGDR/eGDRT, Age, Sex, and Race/Ethnicity; Model 3: All the covariates.

### Subgroup analysis

Table 3 presents the analysis outcomes of the relationship between eGDR and frailty across various subgroups. When eGDR was a continuous variable, the association relationship between eGDR and frailty was significant in males (OR = 0.80; 95% CI: 0.70–0.91), females (OR = 0.83; 95% CI: 0.73–0.93), age ≤ 60 years (OR = 0.73; 95% CI: 0.65–0.82), age > 60 years (OR = 0.88; 95% CI: 0.78–0.99), normal BMI (OR = 0.72; 95% CI: 0.62–0.85), high BMI (OR = 0.78; 95% CI: 0.73–0.84), never smokers (OR = 0.82; 95% CI: 0.73–0.92), former smokers (OR = 0.80; 95% CI: 0.68–0.94), never drinkers (OR = 0.79; 95% CI: 0.66–0.96), former drinkers (OR = 0.83; 95% CI: 0.70–0.98), and heavy drinkers (OR = 0.69; 95% CI: 0.57–0.84). An interaction was observed for alcohol intake (p for interaction = 0.002).

**Table 3:** Tertile stratified subgroup analysis.

Subgroup	eGDR			Tertile of eGDR						
	OR(95% CI)	P value	P for interaction	T1	T2		T3		p for trend (character 2 integer)	P for interaction
					OR(95% CI)	Pvalue	OR(95% CI)	P value		
Sex			0.82							1
Male	0.80(0.70,0.91)	0.001		ref	0.93(0.65,1.34)	0.71	0.86(0.49,1.50)	0.59	0.59	
Female	0.83(0.73,0.93)	0.002		ref	0.82(0.62,1.10)	0.18	0.59(0.38,0.93)	0.02	0.03	
Age			0.03							0.09
=<60	0.73(0.65, 0.82)	<0.0001		ref	0.73(0.53, 1.00)	0.05	0.46(0.28, 0.74)	0.002	0.001	
>60	0.88(0.78,0.99)	0.04		ref	0.95(0.68,1.33)	0.78	0.58(0.30,1.11)	0.1	0.26	
BMI			0.51							< 0.001
Normal	0.72(0.62,0.85)	<0.0001		ref	0.97(0.68,1.40)	0.88	0.52(0.30,0.88)	0.02	0.01	
Low	0.94(0.80,1.12)	0.49		ref	0.89(0.59, 1.35)	0.59	1.34(0.46, 3.93)	0.59	0.93	
High	0.78(0.73,0.84)	<0.0001		ref	0.41(0.27,0.63)	<0.0001	0.52(0.26,1.03)	0.06	0.003	
Smoking			0.35							0.7
Never	0.82(0.73,0.92)	<0.001		ref	0.81(0.60,1.10)	0.18	0.62(0.39,0.98)	0.04	0.04	
Former	0.80(0.68,0.94)	0.01		ref	0.95(0.66,1.36)	0.78	0.79(0.42,1.50)	0.47	0.49	
Now	0.84(0.69, 1.02)	0.08		ref	0.81(0.50, 1.29)	0.36	0.64(0.30, 1.36)	0.24	0.24	
Alcohol intake			0.002							0.005
Never	0.79(0.66,0.96)	0.02		ref	1.08(0.58,2.03)	0.8	0.85(0.31,2.32)	0.75	0.79	
Former	0.83(0.70,0.98)	0.03		ref	1.15(0.71,1.85)	0.58	1.08(0.49,2.41)	0.84	0.82	
Mild	0.87(0.75,1.01)	0.07		ref	0.91(0.61,1.36)	0.64	0.80(0.43,1.48)	0.48	0.48	
Moderate	0.84(0.65, 1.07)	0.15		ref	0.60(0.34, 1.06)	0.08	0.40(0.16, 0.97)	0.04	0.04	
Heavy	0.69(0.57,0.84)	<0.001		ref	0.62(0.36, 1.06)	0.08	0.40(0.17, 0.95)	0.04	0.04	

BMI, body mass index;;T1, 1^st^ tertile; T2, 2nd tertile; T3, 3rd tertile

In addition, according to the tertiles of eGDR results, females (T3 vs T1: OR = 0.59; 95% CI: 0.38–0.93), individuals younger than 60 years (T3 vs T1: OR = 0.46; 95% CI: 0.28–0.74), those with a normal BMI (T3 vs T1: OR = 0.52; 95% CI: 0.30–0.88), never smokers (T3 vs T1: OR = 0.62; 95% CI: 0.39–0.98), moderate drinkers (T3 vs T1: OR = 0.40; 95% CI: 0.16–0.97), and heavy drinkers (T3 vs T1: OR = 0.40; 95% CI: 0.17–0.95) were more likely to reduce the risk of frailty compared with individuals in the first tertile. These subgroup analyses also served as a fairness assessment, showing consistent associations across sex, age, BMI, and lifestyle groups.

### Model Development and Validation

After exclusions, 26,282 participants were included for modeling. VIF and LASSO analyses reduced multicollinearity and identified twelve independent predictors: sex, alcohol use, smoking status, cancer, diabetes mellitus (DM), hypertension, body mass index (BMI), frailty score, systemic immune-inflammation index (SII), estimated glucose disposal rate (eGDR), age, and poverty level (S1 Table and S1 Fig). Class imbalance was addressed using SMOTEN, resulting in a balanced dataset of 41,870 participants, which was split into a training set (n = 29,309) and a test set (n = 12,561). Based on these variables, this study constructed 10 machine learning models and evaluated their performance in predicting frailty on both the testing and validation sets.

**Fig 2:**
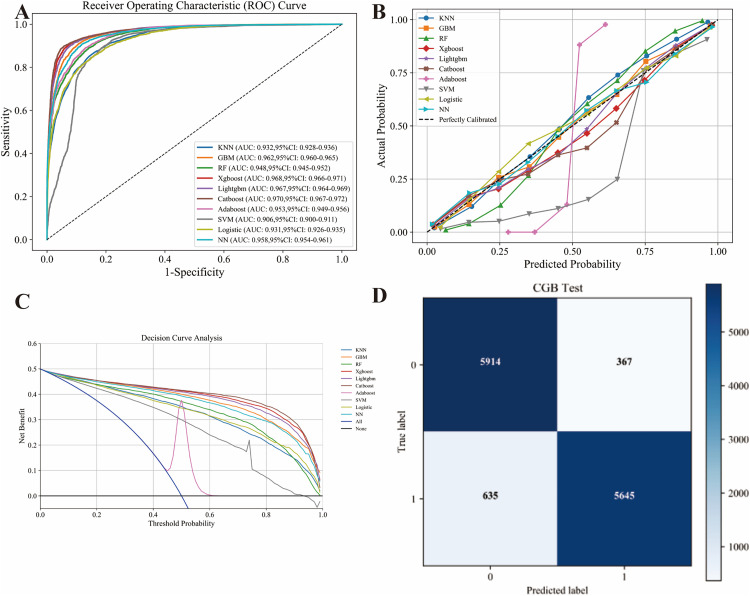
Comprehensive evaluation of machine-learning models in the training cohort. (A) ROC curves and AUC values. (B) Calibration curves. (C) Decision-curve analysis (DCA). (D) Confusion matrix of the optimal CatBoost model in the training set.

Table 4 summarises the ten models built on the testing set. CatBoost achieved the highest AUC (0.970), accuracy (0.920), specificity (0.942), precision (0.939) and F1-score (0.918), followed by XGBoost (AUC 0.968) and LightGBM (AUC 0.967). Fig 2 provides the comprehensive evaluation. Panel A shows the ROC curves: CatBoost’s curve lies furthest to the upper-left, confirming its superior discrimination (AUC 0.970). Panel B displays the calibration plot for CatBoost, which closely follows the diagonal, indicating excellent agreement between predicted probabilities and observed outcomes. Panel C presents the decision-curve analysis: CatBoost confers the highest net benefit across the full range of clinically relevant threshold probabilities. Panel D presents the confusion matrix of CatBoost on the testing set, revealing 5,914 true negatives and 5,645 true positives with only 367 false positives and 635 false negatives, further corroborating its outstanding classification performance.In the independent test set, the CatBoost model maintained stable and robust performance across all metrics. Among the ten models, CatBoost led in AUC and F1 yet demanded careful tuning and offered modest interpretability. Random Forest surrendered slight accuracy for clearer variable influence, whereas logistic regression and KNN trained fastest but fell short in discrimination.

**Table 4:** Performance comparison of the machine learning models in the training cohort (n = 29,309).

Model	AUC	Accuracy	Specificity	Precision	NPV	Recall	F1 score
KNN	0.932	0.854	0.846	0.848	0.86	0.863	0.855
GBM	0.962	0.901	0.891	0.893	0.909	0.911	0.902
RF	0.948	0.874	0.859	0.863	0.886	0.889	0.876
Xgboost	0.968	0.915	0.928	0.926	0.905	0.903	0.914
Lightgbm	0.967	0.911	0.921	0.919	0.903	0.902	0.91
Catboost	0.97	0.92	0.942	0.939	0.903	0.899	0.918
Adaboost	0.953	0.876	0.884	0.882	0.87	0.868	0.875
SVM	0.906	0.853	0.783	0.809	0.91	0.923	0.862
Logistic	0.931	0.848	0.84	0.843	0.853	0.855	0.849
NN	0.958	0.894	0.881	0.884	0.905	0.908	0.896

**Fig 3:**
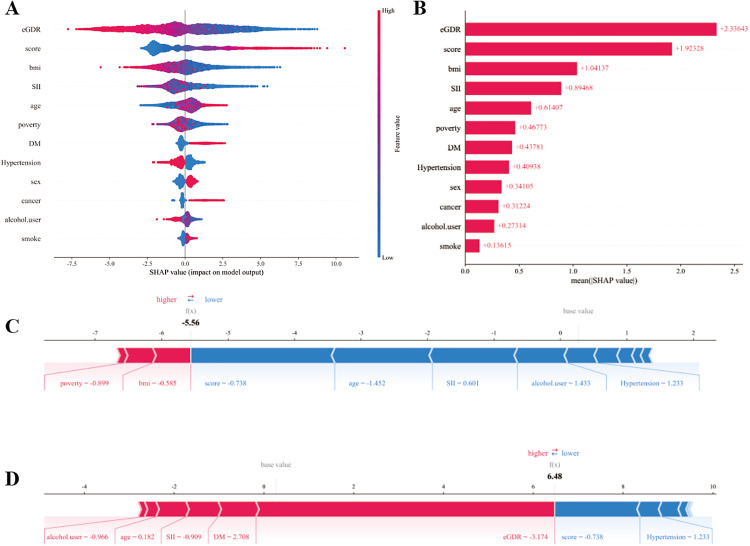
SHAP interpretation of the optimal machine learning model. (A) SHAP summary plot of all predictors; (B) mean SHAP values ranking feature importance; (C–D) SHAP force plots illustrating representative individual predictions. eGDR emerged as the most influential protective factor for frailty.

SHAP analyses were applied to the CatBoost model to visualize feature contributions at both the global and individual levels (Fig 3). The summary plot (Fig 3A) demonstrated that eGDR, frailty score, and BMI were the most influential predictors of frailty, with higher eGDR values strongly associated with a decreased predicted probability of frailty. The bar plot (Fig 3B) confirmed this ranking, with eGDR contributing the largest mean SHAP value. Force plots (Fig 3C–3D) illustrated representative individual predictions, showing how combinations of clinical features either increased (red) or decreased (blue) frailty risk. SHAP plots (waterfall, decision, dependence, and interaction) (S2–S5 Figs)echo these findings: waterfall highlights eGDR, DM, and hypertension as the top drivers in representative cases; decision lines for 100 sampled individuals shift downward with rising eGDR; dependence and interaction plots show a near-monotonic negative effect of eGDR with minimal interaction from BMI or age. Overall, SHAP interpretation highlighted eGDR as the most protective factor, while frailty score and BMI acted as major risk enhancers.

### Mediation analysis of DM between eGDR and frailty

**Fig 4:**
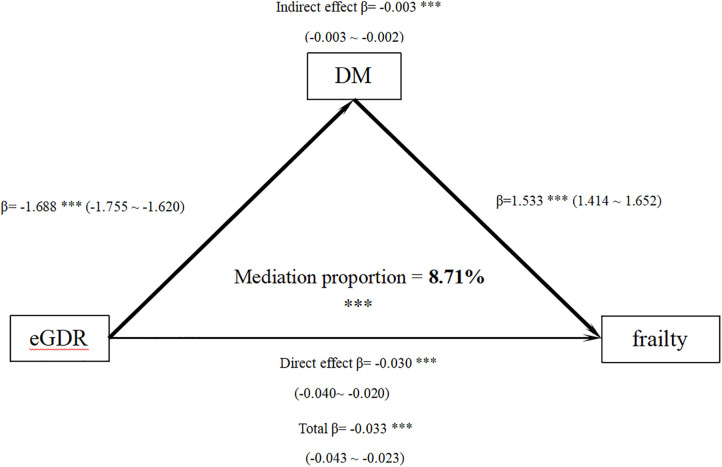
Mediation model illustrating the relationship between eGDR, diabetes, and frailty. Notes: ***P-value < 0.001. 95% CI in the parentheses are shown. Models adjust: age, gender, race, education level, poverty status, marital status, BMI, DM, hypertension, cancer, drinking habits, and smoking status, Physical activity levels, SII.

Bootstrap method to assess the mediating effect of DM. The results showed that the total effect of eGDR on frailty was significant (β= −0.033, 95% CI −0.043 to −0.023, P < 0.001); of which the indirect effect mediated by DM was β= −0.003 (95% CI −0.003 to −0.002, P < 0.001), with a mediation proportion of 8.71% (Fig 4). These findings suggest that DM plays a partial mediating role between eGDR and frailty.

## Discussion

In this prospective cohort study based on the NHANES public database, we included a large, multi-ethnic sample to explore the relationship between eGDR and frailty. The results revealed a significant negative correlation between eGDR and frailty, which remained significant after rigorous adjustment and was consistent across various population subgroups. We employed ML to construct clinical prediction models for frailty and ultimately determined that the CatBoost model performed the best through performance evaluation. In the feature importance analysis, eGDR emerged as the most critical predictor in the model. Additionally, we confirmed the mediating role of DM between eGDR and frailty through mediation analysis. To our knowledge, this study is the first to validate the significant negative correlation between eGDR and frailty using NHANES data and to develop machine learning – based prediction models that explore the mediating role of DM. These findings may assist in the management and intervention of frailty and aid in clinical decision – making.

Several studies have provided solid scientific evidence of the association between IR and frailty. Research has indicated a correlation between elevated IR levels and higher rates of frailty and cardiovascular risk, highlighting the importance of early intervention in young adults with abnormal glucose metabolism [12]. Furthermore, studies have demonstrated a link between metabolic syndrome, IR, and a heightened risk of frailty in the elderly population [11]. As an indicator of IR, HOMA-IR has been validated to correlate with frailty among middle-aged and older adults in the United States [11,16]. Furthermore, data from NHANES 1999–2018 compared different IR indices with frailty, further confirming the association between the two elements [24]. These studies consistently support IR as a key factor in frailty and its close association with cardiovascular disease risk. However, as a new assessment index for IR, eGDR has not been studied for its relationship with frailty.

As economic levels and quality of life have improved, IR among pre-diabetic adults in the United States is on the rise [25,26]. In this context, eGDR, as a new indicator for evaluating insulin resistance, is closely associated with various disease health outcomes [10,27,28]. Compared to previous studies, eGDR has shown unique advantages in assessing IR and frailty risk. Specifically, eGDR is based on the calculation method of waist circumference, hypertension and HbA1c, which is not only easy to implement, but also suitable for screening in large-scale populations [10]. Compared with other IR indicators such as METS-IR, HOMA-IR and TyG, there is a stable negative correlation between eGDR and frailty risk [24]. That is, with the increase of eGDR value, the frailty risk of individuals decreases. This linear relationship makes eGDR more simple and intuitive in analysis, while HOMA-IR and TYG may need more complex nonlinear models to describe their relationship with weakness.Moreover, eGDR does not require to measure fasting blood glucose or insulin levels, which makes it particularly useful in populations where these measurements are difficult to obtain. Compared to METS-IR, eGDR is simpler to operate, so it has more advantages in clinical application, helping to identify the risk of weakness earlier and take intervention measures. To sum up, as a novel IR assessment tool, eGDR has clear advantages in predicting frailty risk compared to traditional indicators, providing a new perspective for future research and clinical practice.

IR is strongly linked to a heightened risk of cardiovascular disease (CVD), which involves pathological processes, such as changes in lipid metabolism, inflammation, endothelial dysfunction, and atherosclerosis [29–31]. Characteristics of frailty syndrome, such as muscle weakness and reduced exercise tolerance, are closely related to the occurrence and development of CVD [32,33]. Therefore, it can be reasonably speculated that IR may indirectly affect frailty status by promoting the development of CVD. The novel indicator eGDR for IR has been identified as being linked to a reduced risk of CVD, indicating that it may slow the development of frailty by reducing CVD risk [10]. In addition, there is a negative association between eGDR and muscle loss, which is a key component of frailty [34]. Therefore, eGDR may be associated with the occurrence and development of frailty through pathways, such as influencing muscle loss, inflammatory responses, and endothelial dysfunction. These findings further highlight the value of eGDR as a non-insulin-dependent IR assessment tool, which is easy to obtain from clinical data and has good stability.

While this study has made progress in exploring the relationship between eGDR and frailty and the mediating role of DM, several limitations remain. First, the cross-sectional design of this study limits our ability to determine the causality between eGDR and frailty. Future research should employ longitudinal designs to further investigate the dynamic relationship between eGDR and frailty. Second, although we considered various potential confounding factors, this study may still be subject to certain biases. For example, genetic, environmental, and lifestyle factors, which were not included in the analysis, may significantly impact the likelihood of frailty [35,36]. Therefore, future studies should incorporate a broader range of these factors to enhance the accuracy and robustness of the findings.In terms of machine learning model development and evaluation, while the CatBoost model performed exceptionally well, its performance may be influenced by sample characteristics and data quality. Future research could explore additional machine learning algorithms, such as deep learning models, to further improve predictive accuracy and generalizability. Moreover, model interpretability is an important area for future research. Although the CatBoost model identified eGDR as a key predictor in feature importance analysis, translating these features into clinically actionable recommendations requires further investigation. Regarding mediation analysis, while we confirmed the mediating role of DM between eGDR and frailty, the magnitude and direction of the mediating effect may be influenced by various factors. Future research could further explore other potential mediating variables, such as inflammatory markers and psychosocial factors, to gain a more comprehensive understanding of the complex relationship between eGDR and frailty.

Additionally, although NHANES uses a multistage probability design that reasonably represents the U.S. population, geographic and demographic constraints caution against direct extrapolation to low- and middle-income countries or to health-care systems with different structures. Self-reported smoking and alcohol data may introduce recall or reporting bias, likely leading to attenuated or spurious associations and potentially underestimating the true effect of these exposures on frailty risk. The frailty index was built from NHANES variables, yet the absence of an internationally standardised definition—owing to variable item selection and cut-offs—limits cross-study comparability. Although the machine-learning models performed strongly in internal validation, their clinical utility awaits prospective validation. Model choice should balance predictive power with interpretability: tree-based ensemble methods (CatBoost, XGBoost, LightGBM) excelled in discrimination but sacrificed transparency, whereas logistic regression offered the reverse trade-off. Future work in broader populations and diverse datasets is needed to confirm model robustness and enhance external validity for frailty prevention and intervention across heterogeneous health-care contexts.

## Conclusions

This study is the first to use NHANES data to elucidate the significant negative correlation between eGDR and frailty, and to investigate the mediating effect of DM. The results confirm a significant negative correlation between eGDR and frailty, with DM acting as a partial mediator. Subgroup analyses show that this relationship remains stable across different populations. The CatBoost model performs exceptionally well, offering potential for early risk assessment and timely intervention of frailty.

## Supporting information

S1 Fig LASSO regression visualization plot. (TIF)

S2 Fig SHAP decision plot. (TIF)

S3 Fig SHAP dependence plots with interaction coloring. (TIF)

S4 Fig SHAP dependence plots. (TIF)

S5 Fig SHAP waterfall plot. (TIF)

S1 Table Calculation and Measurement Protocols for Estimated Glucose Disposal Rate (eGDR) in NHANES. (PDF)

S2 Table Variance Inflation Factor results. (PDF)

## Abbreviations

NHANES: National Health and Nutrition Examination Survey
IR: Insulin resistance
eGDR: estimated glucose disposal rate
FI: frailty index
DM: Diabetes Mellitus
ML: machine learning
AUC: area under the ROC curve
DCA: decision curve analysis
BMI: Body mass index
HbA1c: glycated hemoglobin
WC: waist circumference
HT: hypertension
SD: standard deviation
IQR: , interquartile range
OR: Odds ratio
CI: Confidence interval
HOMA-IR: Homeostatic model assessment for insulin resistance
METS-IR: Metabolic Score for Insulin Resistance
TyG: Triglyceride-glucose
CVD: cardiovascular disease.
