# Supplementary figures and images for: Estimated glucose disposal rate predicts frailty through diabetes: Evidence from machine learning and mediation models in NHANES
Source: PLoS One. 2025 Oct 7;20(10):e0333388. doi: 10.1371/journal.pone.0333388 (PMC12503301; doi:10.1371/journal.pone.0333388)

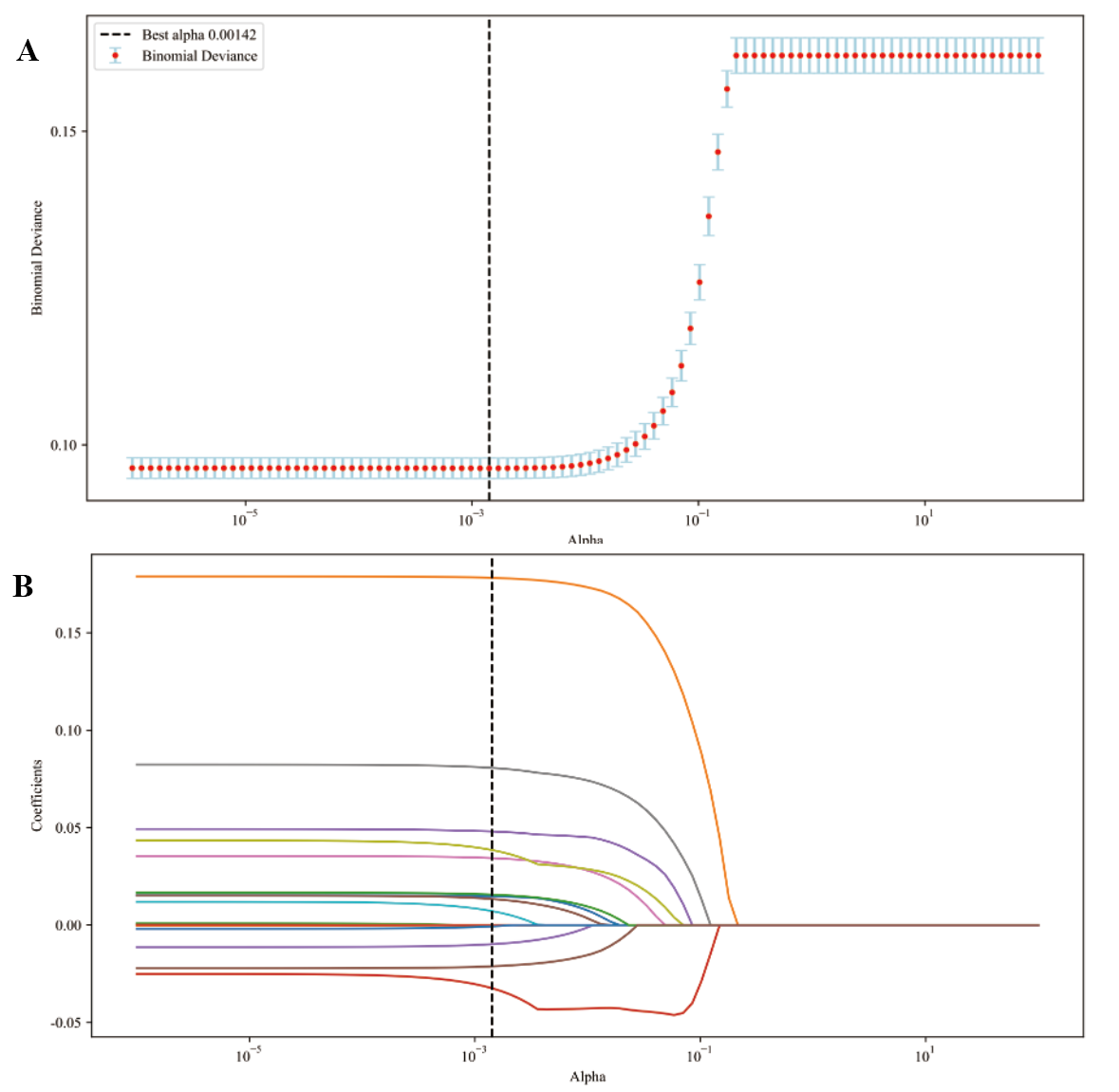

Supplement: S1 Fig — (TIF) [file pone.0333388.s001.tif]

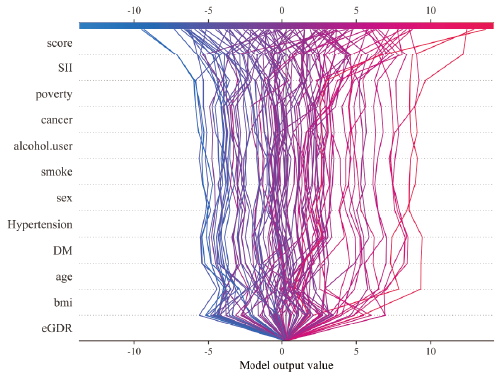

Supplement: S2 Fig — (TIF) [file pone.0333388.s002.tif]

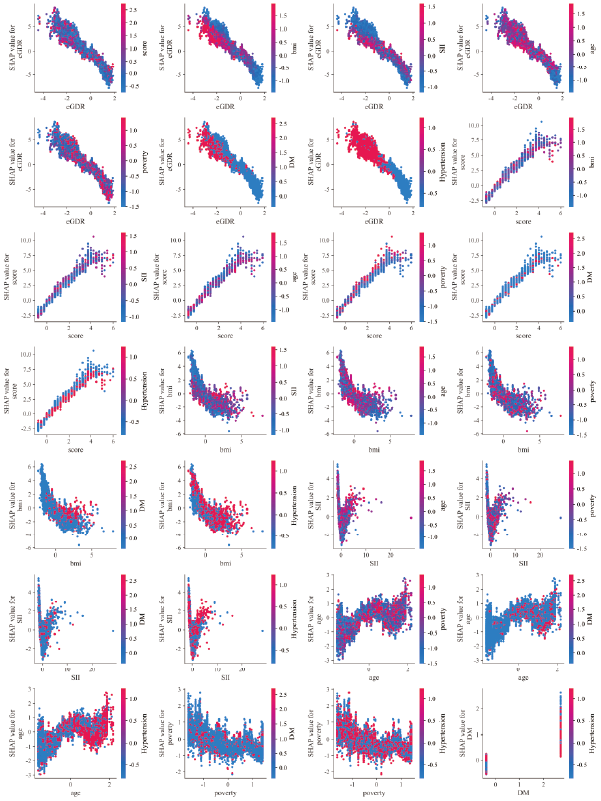

Supplement: S3 Fig — (TIF) [file pone.0333388.s003.tif]

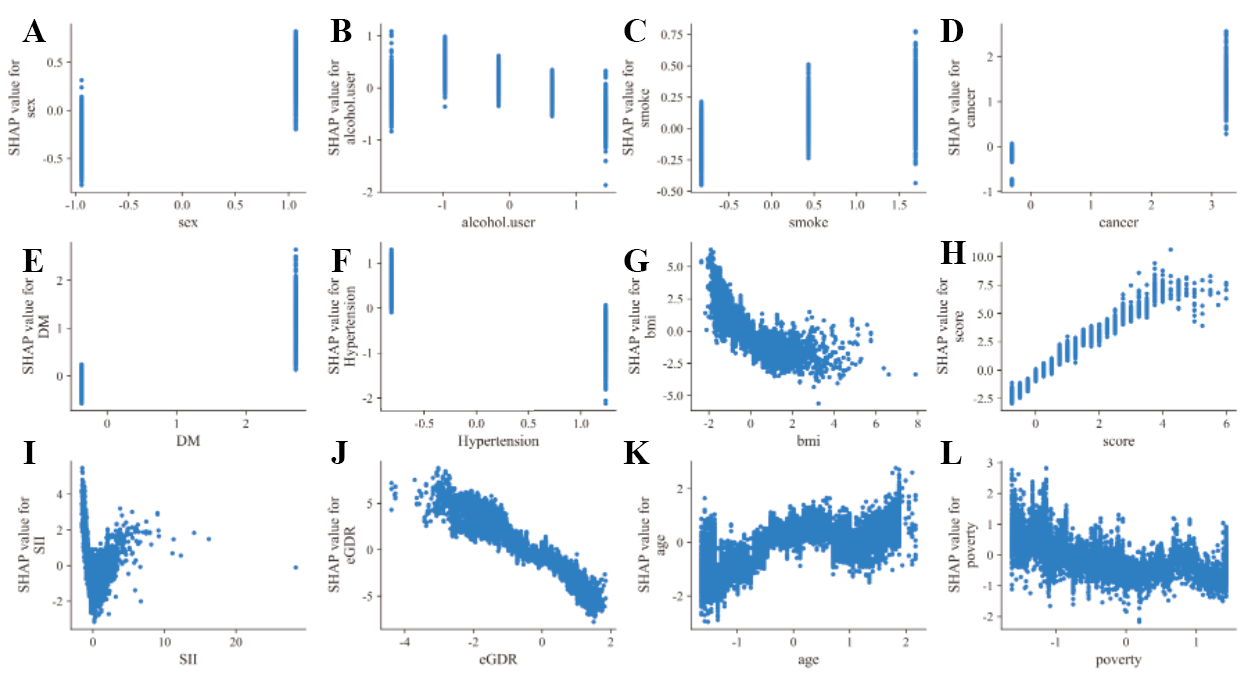

Supplement: S4 Fig — (TIF) [file pone.0333388.s004.tif]

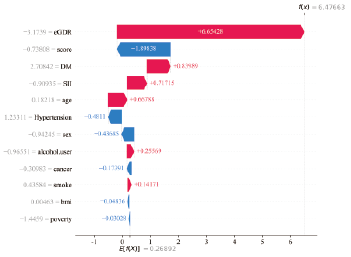

Supplement: S5 Fig — (TIF) [file pone.0333388.s005.tif]
