# Supplementary material for: Estimated glucose disposal rate predicts frailty through diabetes: Evidence from machine learning and mediation models in NHANES
Source: PLoS One. 2025 Oct 7;20(10):e0333388. doi: 10.1371/journal.pone.0333388 (PMC12503301; doi:10.1371/journal.pone.0333388)
Supplement: S1 Table — (PDF) [file pone.0333388.s006.pdf]

eGDR is a novel indicator used to assess IR. We utilized readily available clinical parameters from the NHANES database to evaluate eGDR, such as glycated hemoglobin (HbA1c), blood pressure status (hypertension or normal blood pressure), and waist circumference (WC). The specific calculation formula is: eGDR = 21.158 − (0.09 × WC) − (3.407 × HT) − (0.551 × HbA1c), where WC represents waist circumference (in centimeters), HT indicates hypertension status (yes = 1, no = 0), and HbA1c denotes the percentage of glycated hemoglobin.

Waist measurement method:
1. Position the SP: Instruct the SP to gather the shirt gown above the waist and clip it on the front so it will not interfere with the measurements. Instruct the participant to cross his or her arms and place his or her hands on opposite shoulders. Demonstrate the desired position of the arms. It may help to tell SPs to think of giving themselves a hug. Feel the SP's right and left iliac crest and, if necessary, lower the waistband of the pants and underclothing to expose the iliac crest. Again, always tell the SP what you are going to do before you do it.
2. Mark the measurement site: Stand on the participant's right side. Palpate the hip area to locate the right ilium of the pelvis. With the cosmetic pencil draw a horizontal line just above the uppermost lateral border of the right ilium. Cross this mark at the midaxillary line, which extends from the armpit down the side of the torso.
3. Take the measurement: Extend the measuring tape around the waist. Position the tape in a horizontal plane at the level of the measurement mark (Exhibit 3-19). Use the wall mirror to ensure the horizontal alignment of the tape. While the examiner remains on the SP's right side, the recorder will come around to the SPs left side to check the placement of the tape. Check that the tape sits parallel to the floor and lies snug but does not compress the skin. Always position the zero end of the tape below the section containing the measurement value. Take the measurement to the nearest 0.1 cm at the end of the SP's normal expiration.
4. Record the result: Call the result to the recorder, who will enter this number on the ISIS screen. Remove the tape measure and erase the cosmetic pencil mark from the SP's skin with a few drops of baby oil on a piece of gauze.

Blood pressure status (hypertension or normal blood pressure):
average blood presure was calculated by the following protocol:
The diastolic reading with zero is not used to calculate the diastolic average.
If all diastolic readings were zero, then the average would be zero.
If only one blood pressure reading was obtained, that reading is the average.
If there is more than one blood pressure reading, the first reading is always excluded from the average.

HbA1c method:
In this assay, the stable (SA1c) and labile (LA1c) A1c forms can be individually resolved on the chromatogram without manual pretreatment, allowing accurate measurement of the stable form of HbA1c. The analyzer dilutes the whole blood

specimen with a hemolysis solution, and then injects a small volume of the treated specimen onto the HPLC analytical column. Separation is achieved by utilizing differences in ionic interactions between the cation exchange group on the column resin surface and the hemoglobin components. The hemoglobin fractions (A1c, A1b, F, LA1c, SA1c, A0 and H-Var) are subsequently removed from the column material by step-wise elution using elution buffers each with a different salt concentration. The separated hemoglobin components pass through the photometer flow cell where the analyzer measures changes in absorbance at 415 nm. The analyzer integrates and reduces the raw data, and then calculates the relative percentages of each hemoglobin fraction. Analysis requires three minutes. If a specimen showed a deterioration peak, hemoglobin variant, or a LA1c results $\geqslant$5% and/or LA1c results > half SA1c during the regular test, it would be retested by a second method, ultra 2 HPLC. In the 2017-2018 cycle, only 2.2% of the blood specimens required to be retested by the ultra 2 HPLC method. A lab instrumentation change was made for this secondary method during the data collection period. This instrument change did not significantly affect the resulted glycohemoglobin values; the mean relative error between results from the two instruments was 0.5% (ranged from -4.0% to 5.2%).
