# Supplementary material for: Estimated glucose disposal rate predicts frailty through diabetes: Evidence from machine learning and mediation models in NHANES
Source: PLoS One. 2025 Oct 7;20(10):e0333388. doi: 10.1371/journal.pone.0333388 (PMC12503301; doi:10.1371/journal.pone.0333388)
Supplement: S2 Table — (PDF) [file pone.0333388.s007.pdf]

Table 2 Variance Inflation Factor results.

Variable	VIF
Age	1.46
Sex	1.15
Race/Ethnicity	1.05
Marital status	1.05
Poverty	1.16
Education	1.07
Alcohol intake	1.05
Smoke	1.09
BMI	2.13
Physical Activity	1.04
Cancer	1.07
Diabetes	1.21
Hypertension	2.44
Depression	1.17
SII	1.02
